# Differential Innate Immune Responses Elicited by Nipah Virus and Cedar Virus Correlate with Disparate In Vivo Pathogenesis in Hamsters

**DOI:** 10.3390/v11030291

**Published:** 2019-03-22

**Authors:** Tony Schountz, Corey Campbell, Kaitlyn Wagner, Joel Rovnak, Cynthia Martellaro, Blair L DeBuysscher, Heinz Feldmann, Joseph Prescott

**Affiliations:** 1 Arthropod-borne and Infectious Diseases Laboratory, Colorado State University, Fort Collins, CO 80523, USA; corey.campbell@colostate.edu (C.C.); miedkait@rams.colostate.edu (K.W.); joel.rovnak@colostate.edu (J.R.); 2 Department of Microbiology, Immunology and Pathology, College of Veterinary Medicine and Biomedical Sciences, Colorado State University, Fort Collins, CO 80523, USA; 3 Laboratory of Virology, Rocky Mountain Laboratories, NIAID, Hamilton, MT 59840, USA; cynthia.martellaro@nih.gov (C.M.); bdebuyss@fredhutch.org (B.L.D.); feldmannh@niaid.nih.gov (H.F.); 4 Vaccine and Infectious Disease Division, Fred Hutchinson Cancer Research Center, Seattle, WA 98109, USA; 5 Center for Biological Threats and Special Pathogens, Robert Koch Institute, 13353 Berlin, Germany

**Keywords:** Cedar virus, henipavirus, paramyxovirus, bat virus, Syrian hamster, antiviral response

## Abstract

Syrian hamsters (*Mesocricetus auratus*) are a pathogenesis model for the Nipah virus (NiV), and we sought to determine if they are also susceptible to the Cedar virus (CedPV). Following intranasal inoculation with CedPV, virus replication occurred in the lungs and spleens of infected hamsters, a neutralizing antibody was produced in some hamsters within 8 days post-challenge, and no conspicuous signs of disease occurred. CedPV replicated to a similar magnitude as NiV-Bangladesh in type I IFN-deficient BHK-21 Syrian hamster fibroblasts but replicated 4 logs lower in type I IFN-competent primary Syrian hamster and human pulmonary endothelial cells, a principal target of henipaviruses. The coinfection of these cells with CedPV and NiV failed to rescue CedPV titers and did not diminish NiV titers, suggesting the replication machinery is virus-specific. Type I IFN response transcripts *Ifna7*, *Ddx58*, *Stat1*, *Stat2*, *Ccl5*, *Cxcl10*, *Isg20*, *Irf7*, and *Iigp1* were all significantly elevated in CedPV-infected hamster endothelial cells, whereas *Ifna7* and *Iigp1* expression were significantly repressed during NiV infection. These results are consistent with the hypothesis that CedPV’s inability to counter the host type I IFN response may, in part, contribute to its lack of pathogenicity. Because NiV causes a fatal disease in Syrian hamsters with similarities to human disease, this model will provide valuable information about the pathogenic mechanisms of henipaviruses.

## 1. Introduction

Henipaviruses are negative stranded viruses that belong to the *Paramyxoviridae* family and are hosted by several species of pteropid fruit bats [1,2]. Two henipaviruses, Hendra virus (HeV) and Nipah virus (NiV), cause respiratory disease and encephalopathies with high case-fatality rates in humans and are classified as biosafety level 4 (BSL-4) pathogens [3,4]. HeV was identified and isolated in the 1990s after the severe disease in horses and equine workers, and subsequent work identified pteropus bats (*Pteropus alecto* and *P. conspicillatus*) as reservoir hosts of the virus [5]. NiV was first identified in Malaysia during an outbreak of severe disease amongst abattoir workers that had contracted the virus from swine. Field studies subsequently identified that other species of pteropus bats were reservoir hosts of NiV [6]. Periodic outbreaks of henipavirus disease continue to occur in Australia and Southeast Asia. Since the discovery of these viruses, henipavirus sequences have been detected in many pteropid fruit bat species in Australia, Asia, and Africa, posing a significant risk to humans and livestock.

During the processing of pooled urine samples collected from pteropus bats, a novel henipavirus, Cedar virus (CedPV), was isolated in a cell culture [7]. Sequencing the CedPV genome indicated that its phosphoprotein gene (P) lacked the RNA editing site commonly found in other paramyxovirus genomes, including HeV and NiV, that results in the expression of the V and W accessory proteins [7]. The V and W proteins have been shown to have IFN-antagonism capabilities in cells infected with NiV [8]. CedPV infection does not result in disease in guinea pigs or ferrets, both of which are commonly used in henipavirus pathogenesis studies [7].

Pathogenic henipaviruses infect many cell types, and the targeting and dysregulation of vascular endothelial cells is a major cause of pathogenesis [9,10]. These viruses bind ephrin B2 and ephrin B3 expressed on various subsets of endothelial cells, and the spatial expression of these receptors is associated with viral tropism [11,12]. Ephrin B2 is expressed on cells throughout the body; however, ephrin B3 is primarily expressed on cells in the CNS, and this expression pattern is likely responsible for the CNS tropism of NiV and HeV. CedPV binds to ephrin B2 but not ephrin B3, which may account, in part, for its lack of pathogenicity in rodent animal models [7,13].

NiV and HeV infections cause a fatal disease in Syrian golden hamsters (*Mesocricetus auratus*) with pathogenesis involving the respiratory tract and nervous system similar to that of the human disease [14,15]. We, thus, sought to determine if Syrian hamsters are susceptible to CedPV, whether there are differences in viral replication and immune activation between NiV and CedPV in vitro, and whether these were attributable to CedPV’s inability to cause disease. We compared the immune responses to infection with NiV and CedPV in a cell line deficient in IFN responses and in primary endothelial cells. Both human and hamster primary endothelial cells, the targets of henipavirus infection, generated IFN responses to CedPV that were not observed upon NiV infection. The infection of hamsters by NiV results in severe disease; however, CedPV infection resulted in an immune response that cleared the virus without disease. Together, these data suggest that CedPV activates innate immune responses and is unlikely to cause disease in an immunocompetent host, which is likely due, in part, to the lack of V and W accessory proteins.

## 2. Materials and Methods

### 2.1. Ethics Statement

The Institutional Animal Care and Use Committee of the Rocky Mountain Laboratories approved all animal experiments, which were performed following the guidelines of the Association for Assessment and Accreditation of Laboratory Animal Care, International (AAALAC) by certified staff in an AAALAC-approved facility (protocol 2013-059E). All infectious virus work was conducted in the biosafety level 4 (BSL-4) laboratory at Rocky Mountain Laboratories, NIAID (Hamilton, MT, USA) following established BSL-4 standard operating procedures that were approved by the Institutional Biosafety Committee. The isolation of primary microvascular endothelial cells was approved by the Colorado State University Institutional Animal Care and Use Committee (14–4976A, 25/04/2014).

### 2.2. Viruses and Cells

The Bangladeshi strain of the Nipah virus (NIV) was obtained from the Viral Special Pathogens Branch of the CDC, was originally isolated from a fatal human infection in Bangladesh in 2004, and was passaged in Vero E6 cells a total of three times [16]. Cedar virus (CedPV) was obtained from CSIRO (Geelong, Australia) and was isolated from bat urine inoculated onto primary bat kidney cells, followed by passaging on Vero E6 cells (ATCC) [7]. Both viruses were propagated on Vero E6 cells at RML in Dulbecco’s minimal essential medium (DMEM) (Sigma) supplemented with 10% fetal bovine serum, 2 mM L-glutamine, 50 IU/mL penicillin, and 50 µg/mL streptomycin (Life Technologies, Carlsbad, CA, USA).

BHK-21 cells (CCL-10, ATCC, Manassas VA, USA) were propagated in 24-well tissue culture plates and inoculated with CedPV or NiV (or mock) with a MOI of 1.0 or 0.1 for 1 h, washed twice in DMEM, and incubated at 37 °C, 5% CO_2_ for the indicated amount of time.

Human lung blood microvascular endothelial cells (LBMVEC) (CC-2527, Lonza, Walkersville MD, USA) were maintained in an EGM-2 medium (Lonza). The cells were seeded in 24-well plates one day prior to inoculation with CedPV or NiV with a MOI of 0.1. At the indicated time points, supernatants were collected for virus quantitation and the cells were lysed in buffer RLT (Qiagen) to quantitate viral or cellular RNA.

### 2.3. Hamster Infections

Five- to six-week-old female Syrian golden hamsters (Harlan, Indianapolis, IN, USA) were inoculated intraperitoneally (IP) (a total of 500 µL) or intranasally (IN) (a total of 100µL) with 10^5^ TCID_50_ of CedPV that was diluted in sterile DMEM. CedPV was passaged twice on *P. alecto* kidney cells upon original isolation from bats and passaged twice on Vero E6 cells to generate a virus stock. The control animals received DMEM only. At the indicated time points, groups of 4 hamsters were euthanized and the lung tissue was collected for virus quantitation and histology, and sera was collected for the measurement of antibody titers by a neutralization assay.

### 2.4. Virus Quantification

RNA was extracted from homogenized tissue samples using the RNeasy Mini Kit (Qiagen, Carlsbad, CA, USA) following manufacturer’s instructions and according to our established protocols. Viral RNA was quantitated using a one-step real-time RT-PCR targeting NP genes with a Rotor-Gene probe kit (Qiagen) (primer and probe sequences available on request). Standard dilutions of the RNA extracted from the titrated virus stocks were assayed in parallel to calculate the FFU equivalents of viral RNA.

The tissue culture infectious dose 50% (TCID_50_) method was used to quantitate CedPV and NiV in the supernatants of the infected cell cultures. Monolayers of the Vero E6 cells were grown in 96-well plates, and 100 µL of the serial 10-fold diluted samples in MEM containing 2% FBS were added to the wells in triplicate. The cells were then incubated for 5 days at 37 °C with 5% CO_2_ and then scored for CPE, and the Spearman–Karber method was used to calculate the TCID_50_.

### 2.5. Virus Neutralization Assay

The neutralizing antibody titers were measured by a neutralizing tissue culture infectious dose 50% assay (NTCID_50_). For this, 100 TCID_50_ of CedPV was incubated with serial dilutions of hamster sera for 1 h at 37 °C. This was used to inoculate monolayers of the Vero E6 cells in 96-well plates for 1 h. After five days of incubation, the CPE was examined and the NTCID_50_ was calculated using the Spearman–Karber method.

### 2.6. Generation of Primary Hamster Pulmonary Microvascular Endothelial Cells

To obtain pulmonary endothelial cells from Syrian hamsters, the protocol from reference [17] was modified. Briefly, 5-day-old hamster littermates were anesthetized with isoflurane to effect before euthanasia by thoracotomy. Immediately after euthanasia, the lungs were aseptically collected and placed in ice-cold, serum-free DMEM. The lungs were then transferred to sterile Petri dishes, and the excess DMEM was aspirated. Tissues were minced by repeated cutting with sterile scissors approximately 100 times before enzymatic digestion with a collagenase/elastase (C1639, E7885, Sigma, St. Louis, MO USA) cocktail at 37 °C on an orbital rocker for 45 min. The digested cell suspension was passed through a 20 g needle 12 times to generate a single cell suspension before an isolation medium (20% FBS, 1% pen/strep in DMEM) was added to neutralize the enzymatic digestion. After tissue digestion, the cells were pelleted by centrifuging at 400× *g* for 5 min and then washed in PBS containing 0.1% BSA. The cells were resuspended in 100 µL of 0.1% BSA-PBS (Ca^2+^Mg^2+^-free). A magnetic separation was used to select for endothelial cells. Fifty microliters of magnetic beads conjugated to Protein G (G7471, Promega, Madison, WI, USA) were washed 4 times in 0.1% BSA-PBS before resuspension in 0.1% BSA-PBS. The anti-CD31 (PECAM1) antibody (LS-C150165, LSBio, Seattle, WA, USA) was conjugated to the magnetic beads by mixing 50 µL of the antibody with 100 µL of the magnetic bead slurry for 2 h at room temperature on an orbital mixer. The antibody-conjugated beads were then washed twice in 0.1% BSA-PBS before being resuspended in 500 µL of 0.1% BSA-PBS. To sort the cells, 250 µL of the antibody-conjugated beads were added to 1 mL of the resuspended cells and tubes were tumbled on an orbital mixer for 15 min at RT. The samples were then placed on a magnet, and the cells not bound to the magnetic beads were aspirated and discarded. The cells bound to beads were washed 4 times in 0.1% BSA-PBS before being resuspended in 1 mL of the VascuLife EnGS Mv endothelial cell culture media (LL-0004, LifeLine Cell Technology, Carlsbad, CA USA). The cells were plated in 35-mm cell culture dishes that had been pre-coated in gelatin (G1393, Sigma, St. Louis, MO, USA), and an additional 1 mL of media was added. Half of the medium (1 mL) was changed every other day until the cells had grown to 80–90% confluency, at which time the cells were expanded and used in experiments.

### 2.7. Real-Time PCR of Host Response Genes

SYBR Green real-time PCR was performed using primers for a subset of innate response genes (Supplemental Table S1). Briefly, the primers were designed using software developed by us [18] from the Syrian hamster RefSeq RNA database. Primary hamster endothelial cells were infected with 0.1 MOI of CedPV or NiV for 1 h followed by medium replacement (2% FBS-DMEM). The total RNA was extracted from endothelial cells (RNAEasy, Qiagen) at 24 and 48 h postinfection and reverse transcribed (QuantiTech RT, Qiagen) according to manufacturer’s instructions, followed by qPCR (QuantiTech SYBR Green Master Mix, Qiagen) with a 96-well LightCycler (Roche). The cycling parameters were a 15 min activation step followed by 40 cycles of 15 s at 94 °C, 30 s at 56 °C, and 30 s at 72 °C. Melt curves were generated post-amplification to ensure only single-peak products were generated. The relative template abundances were determined using the ΔΔCt method normalized on GAPDH.

## 3. Results

### 3.1. Cedar Virus Infects Hamsters without Signs of Disesae

**Figure 1:**
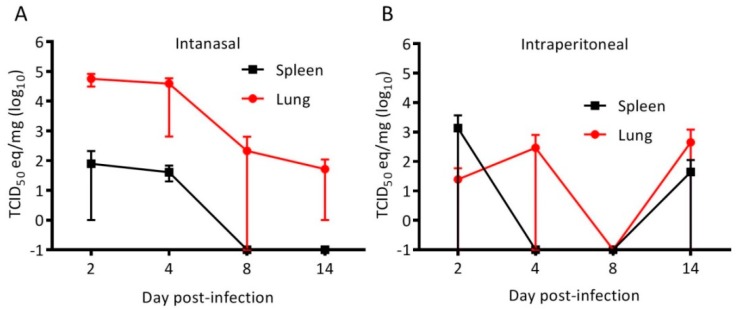
The cedar virus replication in hamsters is more robust via the intranasal route. The hamsters were challenged with 10^5^ TCID_50_ CedPV via the intranasal (**A**) or intraperitoneal (**B**) routes. The tissues were homogenized, and the total RNA was extracted and used to quantitate CedPV viral RNA by qRT-PCR. At each time point, intranasal inoculation resulted in a more robust replication in the lungs, with the exception of day 14. No viral RNA was detected at day 28 postinoculation. The geometric means are plotted and error bars represent the 95% CI.

**Figure 2:**
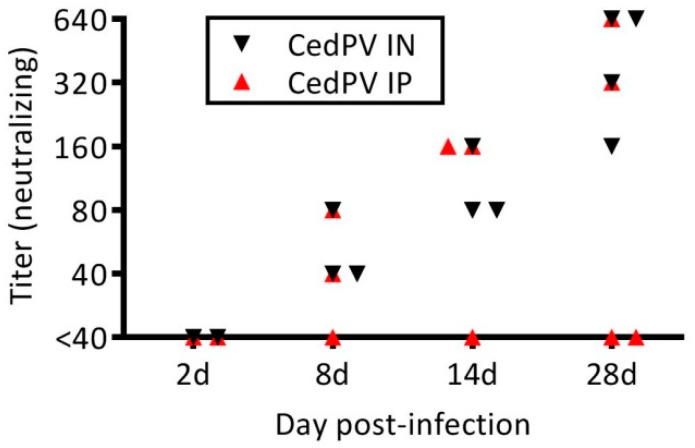
Intranasal inoculation leads to a more frequent seroconversion in hamsters. The hamsters intranasally challenged with CedPV as described in Figure 1 all seroconverted as measured by CedPV neutralization by day 14, whereas only 2 of 4 of those intraperitoneally challenged seroconverted, which had similar titers to intranasally inoculated hamsters.

Hamsters were inoculated with 10^5^ TCID_50_ of CedPV via intraperitoneal (IP) or intranasal (IN) routes to determine route-specific susceptiblity. During the 28-day experiment, none of the hamsters showed conspicuous signs of disease and a gross examination of the organs was unremarkable. Groups of hamsters were euthanized 2, 4, 8, 14, and 28 days postinoculation, and the lungs and spleens were found to have vRNA through day 14, with the execption of day 8 IP-inoculated hamsters, in which none had detectable vRNA by qRT-PCR (Figure 1). Virus isolation was not attempted because, at least for the other henipaviruses, RT-PCR is substantially more sensitive for testing animal tissues. No CedPV was detected at the 28-day time point for either inoculation route. A neutralizing antibody appeared in all but one hamster by day 8. On day 28, all IN-inoculated hamsters had a neutralizing antibody (GMT 381), whereas only 2 of the 4 IP-inoculated hamsters had a neutralizing antibody (titers of 320 and 640) (Figure 2).

### 3.2. Cedar Virus Replicates in Hamster Cells

**Figure 3:**
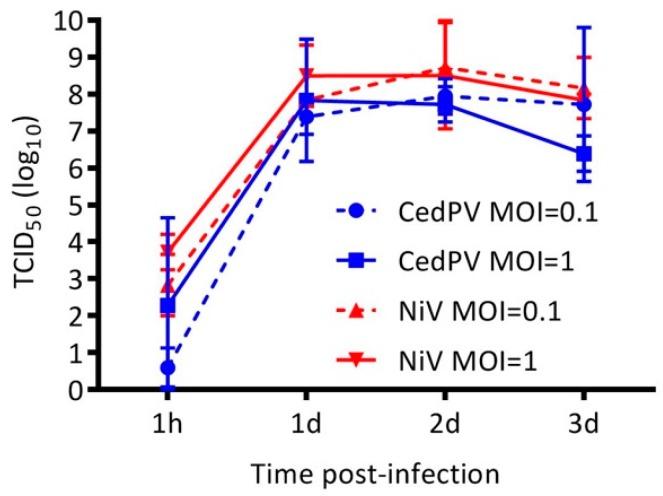
CedPV and NiV replicate similarly in hamster BHK-21 cells. Regardless of MOI, CedPV and NiV replicate with similar kinetics and to similar titers in type I IFN-deficient BHK-21 cells. The data are represented as the geometric mean titers and 95% CI from the titration of the supernatant of infected monolayers of BHK-21 cells. A 2-way ANOVA with Bonferroni’s posttest was used to compare the conditions and showed no significant differences.

**Figure 4:**
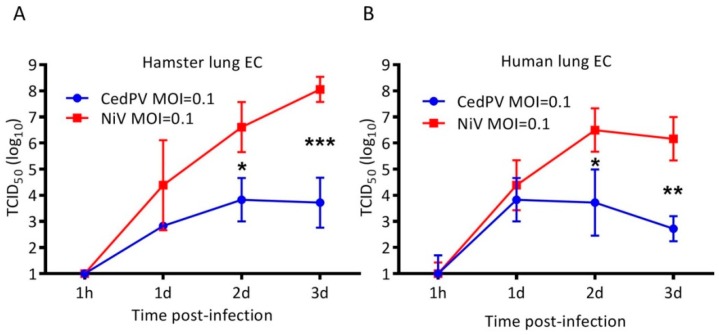
CedPV replicates poorly in primary hamster (**A**) and human (**B**) pulmonary endothelial cells. Monolayers of the cells were inoculated with CedPV or NiV at 0.1 MOI, and the supernatants were sampled for 3 days, at which time NiV replicated to more than 4 logs more than CedPV. The data are represented as the geometric mean titers and 95% CI. A 2-way ANOVA with Bonferroni’s posttest was used to compare the viruses (* *p* < 0.05, ** *p* < 0.01, and *** *p* < 0.001).

Henipaviruses have wide species and cellular tropisms, and we were interested in determining whether the differential replication of CedPV and NiV in hamster cells in vitro could account for their disparate disease-causing potential in vivo. We inoculated the Syrian hamster-derived BHK-21 cells with 0.1 or 1 MOI of CedPV or NiV and found that both viruses replicated to similarly high titers and with similar kinetics (Figure 3). BHK-21 cells are deficient in type I IFN activation [19], and because CedPV lacks V and W innate response antagonist accessory proteins, we hypothesized that cells with intact innate responses would differentially affect CedPV and NiV replication kinetics. We examined both virus’ capacity to infect primary hamster lung endothelial cells, which are major targets of henipaviruses in vivo. We selected for CD31^+^ pulmonary microvascular endothelial cells from 5-day-old Syrian hamster pups and propagated them in an endothelial cell selection medium. In comparison to NiV, which expresses V and W accessory proteins, CedPV replication by day 3 postinfection was more than 4 logs less than NiV (Figure 4A). To assess whether human cells behave similarly, we infected human primary pulmonary endothelial cells with CedPV or NiV (Figure 4B). Similar to what was observed in the hamster cells, NiV replication was approximately 3 to 4 logs higher than CedPV replication after 3 days. By day 3, NiV-infected human endothelial cells were near 100% killed, whereas CedPV-infected endothelial cells appeared similar to the control mock-infected cells.

### 3.3. NiV Fails to Rescue CedPV Replication in Primary Hamster Endothelial Cells

**Figure 5:**
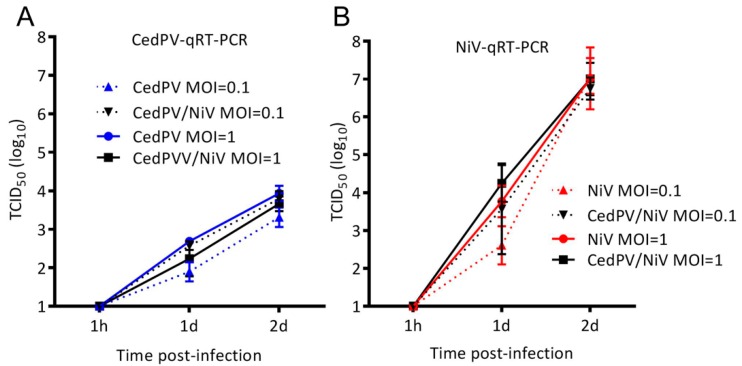
The coinfection of IFN-competent primary hamster endothelial cells does not alter virus replication kinetics. CedPV (**A**) or NiV (**B**) replication was measured by virus-specific qRT-PCR. A similar viral RNA abundance was measured regardless of MOI. The data are represented as the geometric mean titers and 95% CI.

Hamster primary endothelial cells were coinfected with NiV and CedPV to determine whether each virus influences the replication of the other (Figure 5). NiV replication was similarly high regardless of coinfection or MOI, whereas CedPV’s replication in coinfected cells was no more elevated than in endothelial cells only infected with CedPV (Figure 4), suggesting that the polymerase complexes are virus-specific and that repression of the innate response by NiV does not result in increased CedPV replication.

### 3.4. CedPV Fails to Block Antiviral Defenses in Primary Hamster Endothelial Cells

**Figure 6:**
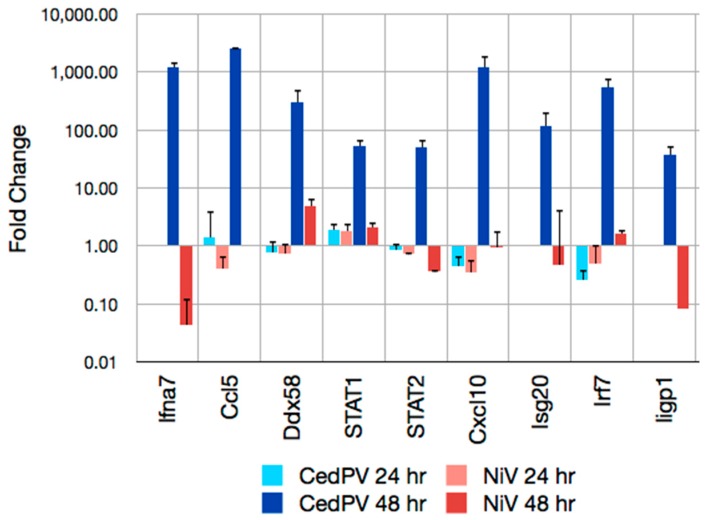
The robust innate gene expression in primary hamster endothelial cells infected with CedPV: Hamster endothelial cells were infected with 0.1 MOI of CedPV or NiV, and the total RNA was extracted at 24 and 48 h. Real-time PCR was performed to determine the fold-change in gene expression. At 24 h, the expression of Ifna7, Isg20, and Iigp1 were not detected. At 48 h, Ccl5 expression was not detected in the NiV-infected cells.

Because of the apparent lack of an RNA editing site in the P gene of CedPV, which encodes V and W proteins of other paramyxoviruses that interfere with innate immune responses, we examined the expression of several innate immune genes in hamster primary endothelial cells infected with CedPV or NiV (Figure 6). At 24 h post-infection, there were no differences in the expression levels of these genes. However, at 48 h, many innate immunity-related genes were elevated in the CedPV-infected endothelial cells, some more than 1000-fold, that were only modestly elevated or repressed in NiV-infected endothelial cells. Two genes, interferon-α 7 (*Ifna7*) and interferon inducible GTPase 1 (*Iigp*), were repressed more than 10-fold in the NiV-infected cultures, suggesting an active repression of these genes by NiV.

## 4. Discussion

Previous work showed that, unlike NiV, CedPV fails to cause disease in ferrets or guinea pigs [7]. Because of the number of immunological reagents and the available annotated genome for Syrian hamsters, which are model organisms that recapitulate many aspects of human disease caused by NiV and HeV, we challenged hamsters with CedPV and determined that they were susceptible to infection but do not develop disease. The inoculation of hamsters indicates the intranasal route is more efficient at inducing infection than is the intraperitoneal route (Figure 1). Despite the lower frequency of infection via the intraperitoneal route, those hamsters that became infected developed neutralizing antibody responses that paralleled those infected intranasally (Figure 2). The replication of CedPV in the lungs of intranasally inoculated hamsters at early time points (2–4 days postinfection) was approximately 10^2^–10^3^ lower than what we observed for NiV at a similar time point (5 days) [20]. Comparisons after this time point are not possible because hamsters succumb to infection between 5–7 days postinfection. To elucidate the innate immune differences between the pathogenic NiV and apathogenic CedPV, we performed cell culture experiments using hamster and human cells. The lack of pathogenicity caused by CedPV, in contrast to NiV/HeV, correlated with the induction of CedPV-induced innate immune responses.

Nearly all paramyxoviruses, including NiV and HeV, possess a phosphoprotein (P) gene that is unusual in that it has an RNA editing site resulting in frameshifts that lead to alternative reading frames that encode three other polypeptides: C, V, and W [21,22,23,24]. However, CedPV is devoid of this RNA editing site and is incapable of producing V or W proteins. The loss of this site appears to be natural and is not a result of passaging in the cell culture because there are no coding regions in the P gene that are similar to V or W of HeV or NiV [13,25]. The V and W proteins interfere with the type I IFN response by disrupting the STAT1 and STAT2 localization to the nucleus [8]. In BHK-21 cells, a fibroblast line incapable of secreting type I IFN [26], both CedPV and NiV replicated with similar kinetics and to similar titers (Figure 3). However, in type I IFN-competent hamster primary cells, CedPV replication was reduced by more than 4 logs compared to NiV and to CedPV in BHK-21 cells (Figure 4), suggesting that CedPV is unable to suppress the type I IFN response, resulting in an antiviral state that tempers replication. This was recapitulated in human primary pulmonary endothelial cells.

We hypothesized that the coinfection of hamster endothelial cells (Figure 5) would rescue CedPV replication to levels found in BHK-21 fibroblast cells (Figure 3) because NiV suppresses the type I IFN response. However, CedPV replication remained at the same level as with virus alone in these cells (compare Figure 4A to Figure 5A). There are two possible explanations for the failure of NiV coinfection to support a greater CedPV replication in primary endothelial cells. First, there were insufficient numbers of dually infected cells with a NiV-mediated block of the innate immune mechanism in CedPV-infected cells. This outcome may be due to insufficient numbers of coinfecting particles or may result from superinfection exclusion [27]. Such an exclusion would effectively prohibit the dual infection of individual cells and prevent the protection of CedPV by NiV. A second possible mechanism is cell type-dependent differences in RNA replication kinetics. The CedPV and NiV polymerase complexes may be viral RNA-specific and rate limiting in endothelial cells. Paramyxovirus polymerase complexes are composed of the polymerase (RdRp), nucleoprotein (N), and the phosphoprotein (P). The nucleoproteins of the CedPV and NiV share only a 66% identity, and the RdRp share only a 57% identity but CedPV’s RdRp is 256 amino acids larger [7]. The P proteins share only a 34% identity with the great majority of diversity between residues 200–400. Thus, there are sufficient differences between the polymerase complexes to suggest that their function would be restricted from the replication of a coinfecting virus *in trans*. This explanation also requires that a restricted function of the CedPV RdRp occurs specifically in primary endothelial cells. However, in BHK-21 cells, a maximal CedPV polymerase activity leads to a high titer replication.

To further scrutinize the impact on the type I IFN response in hamster endothelial cells, we examined the gene expression profiles of several interferon response genes by qPCR. NiV effectively suppressed the expression of these genes, whereas CedPV did not (Figure 6). Together, these data suggest that an inability of CedPV to circumvent the type I IFN response may contribute to its reduced replication and limits its ability to cause disease in hamsters.

Cellular attachment by HeV and NiV is restricted to ephrin B2 and ephrin B3 [7,28]. It is thought that ephrin B3 binding is a prerequisite for encephalitis in humans and other mammals because it is expressed by cells of the brain stem, whereas ephrin B2 is principally found on other cells, including endothelial cells. Because CedPV selectively binds ephrin B2, it is likely unable to infect cells of the brain stem. However, it is unclear whether the combination of type I IFN induction, lower replication, and lack of ephrin B3 tropism leads to an apathogenic transient CedPV infection. Future studies using introduced mutations of the recently described reverse-engineered infectious clone of CedPV [29] will be essential for addressing these questions.

The loss of the V and W proteins in CedPV also raises important biological questions about their roles in the reservoir bat species. The clear evidence that CedPV circulates amongst pteropid bats in Australia [30] implies V and W are dispensable for persistence in bats, some of which have constitutively activated interferon pathways. The loss of V and W proteins should be detrimental to CedPV replication. Its persistence implies the existence of other viral and host factors that contribute to CedPV ecology.

## 5. Conclusions

We have developed a new animal model using the Syrian golden hamster for the study of CedPV infection. The infection fails to cause disease and results in high-titer neutralizing antibody responses and virus clearance. In addition to the previously reported receptor specificity to only ephrin B2 and the lack of V and W genes, we have demonstrated that CedPV cannot suppress the type I IFN response as NiV can, and this may contribute to CedPV’s inability to cause disease. We have determined a third difference of CedPV compared to NiV in that it has poorer replication kinetics that may be associated with its failure to control IFN or with differences in its RNA specificity and/or polymerase complex.

## Supplementary Materials

The following are available online at https://www.mdpi.com/1999-4915/11/3/291/s1, Table S1: Primers for qPCR of hamster immune-related genes.
