# Supplementary material for: Differential Innate Immune Responses Elicited by Nipah Virus and Cedar Virus Correlate with Disparate In Vivo Pathogenesis in Hamsters
Source: Viruses. 2019 Mar 22;11(3):291. doi: 10.3390/v11030291 (PMC6466075; doi:10.3390/v11030291)
Supplement: Supplementary file 1 [file viruses-11-00291-s001.pdf]

Supplemental Data

Table S1. Primers for qPCR of hamster immune-related genes.

Gapdh	TGTGGAAGGACTCATGACCA	GGATGCAGGGATGATGTTCT
Ifna7	CTGGTGGCTGTGAGGAAATA	AGCAAGTTGGCTGAGGAAGA
Ccl5	ACTGCCTCGTGTTCACATCA	CCCACTTCTTCTTTGGGTTG
Ddx58	CGCGGAACTTTGAAGAGAAG	TTGGTCTCCGGCTTTAAGTG
Stat1	CAATATAGCCGCTTTTCTTTGG	TGTACAGGATCCTCCTGGAAGT
Stat2	TGCTGCCAAAACTGGACGA	TCAACCACTGTTCCAGCTGT
Cxcl10	TGCTACACTTTTAGCCTTGTGC	ACCCAGGTAACTCAGAACTGGA
Isg20	TGCAGCATTGTGAACTTCAGTG	GCAGGATCTCTAGTCTGGCTTC
Irf7	CACTATCCGTGGCTACACTCTG	GGTCCTACTCTGTGATGTGCTG
Iigp1	GGTGCAGCAAAAGTTGGAGTAG	TCCTCCAGATAATCCTTTGGCG
